# The complete mitochondrial genome of the eusocial sponge-dwelling snapping shrimp *Synalpheus microneptunus*

**DOI:** 10.1038/s41598-020-64269-w

**Published:** 2020-05-08

**Authors:** Solomon T. C. Chak, Phillip Barden, J. Antonio Baeza

**Affiliations:** 1 Department of Biological Sciences, New Jersey Institute of Technology, Newark, NJ 07102 USA; 2 Department of Biological Sciences, 132 Long Hall, Clemson University, Clemson, SC 29634 USA; 3 Smithsonian Marine Station at Fort Pierce, 701 Seaway Drive, Fort Pierce, Florida 34949 USA; 4 Departamento de Biología Marina, Facultad de Ciencias del Mar, Universidad Católica del Norte, Larrondo 1281, Coquimbo, Chile

**Keywords:** Mitochondrial genome, Sequence annotation, Marine biology, Bioinformatics, Social evolution

## Abstract

In the marine realm, eusociality is only known to have evolved within a clade of sponge-dwelling snapping shrimps in the genus *Synalpheus*. Deciphering the genomic underpinnings of eusociality in these marine shrimps has been limited by the sparse genomic resources in this genus. Here, we report, for a eusocial shrimp *Synalpheus microneptunus*, a complete mitochondrial genome (22X coverage) assembled from short Illumina 150 bp paired-end reads. The 15,603 bp long mitochondrial genome of *S*. *microneptunus* is AT-rich and includes 13 protein-coding genes (PCGs), 2 ribosomal RNA genes, 22 transfer RNA genes and an 834 bp intergenic region assumed to be the D-loop. The gene order is identical to that reported for most caridean shrimps and corresponds to the presumed Pancrustacean ground pattern. All PCGs showed signs of purifying selection, with K_A_/K_S_ <<1 across the whole PCGs and most sliding windows within PCGs. Maximum-likelihood and Bayesian inference phylogenetic analyses of 13 PCGs and 68 terminals supports the monophyly of the Caridea and the family Alpheidae. The complete mitochondrial genome of the eusocial shrimp *Synalpheus microneptunus* will contribute to a better understanding of the selective pressures and rates of molecular evolution in marine eusocial animals.

## Introduction

Sponge-dwelling snapping shrimps in the genus *Synalpheus* (Decapoda: Alpheidae) are the only known clade of marine animals to have evolved eusociality^1,2^, a complex social organization that is best known in terrestrial insects such as ants, bees and termites^3^. Eusocial *Synalpheus* colonies typically consist of a single or a few queens^1^ and up to several hundred non-sterile workers of the two sexes^4,5^. At least nine described species of *Synalpheus* in the West Atlantic ‘gambarelloides’ group are known to be eusocial^1^. These species are characterized by their high reproductive skew, overlapping generations, and, in a few representatives in which behavioral observations have been made, cooperative defense of the host sponge^2,6,7^. *Synalpheus* belonging to the ‘gambarelloides’ clade represent a relatively young lineage that radiated between ~5 and 7 Mya^8^, yet eusociality has independently evolved at least four times in this genus^1^. Communal living, where multiple mating pairs live in the same sponge, has also evolved multiple times in this clade from pair-forming ancestors^9^. The social diversity, short evolutionary history, and similar ecology among *Synalpheus* shrimps make them an ideal group of marine animals to study the evolution of sociality. Moreover, despite a decade-long period of ecological dominance^10^, eusocial *Synalpheus* shrimps have experienced recent population declines^11^. Our knowledge of the biology of eusocial shrimps has increased substantially over the past decades. Yet, genomic resources are scarce in this group^12^, especially when compared to that of social insects. Such a lack of genomic knowledge is limiting our understanding of behavioral innovations in sponge-dwelling snapping shrimps. Therefore, this study focuses on the development of genomic resources that are pivotal to improve our understanding of evolutionary innovations in this and other groups of crustaceans.

*Synalpheus microneptunus* is found only in reefs along the west coast of Barbados in the eastern Caribbean Sea and is the only known eusocial species in Barbados^13^. Their colonies typically consist of <10 individuals with a single ovigerous female (i.e., the queen). They live in sponges belonging to *Neopetrosia proxima* and *N*. *subtriangularis* (previously *Xestospongia*) and may share the sponge host with *S*. *belizensis*, a pair-living species^13^. *S*. *microneptunus* comprised 25% of the total abundance of *Synalpheus* in Barbados, despite being one out of 14 *Synalpheus* species in the region. This observation mirrors the ecological dominance of eusocial species observed in Belizean coral reefs documented by a decade of field survey^10^. *Synalpheus microneptunus* is part of the *S*. *paraneptunus* species complex that shares several synapomorphic features that distinguish them from the rest of the *S*. *gambarelloides* group, including sparse, unorganized setae on the non-snapping minor chela (while most other species have organized rows of setae) and the “excavated” inner surface of the fingers of the minor chela^14^. Phylogenetically, *S*. *microneptunus* is closely related to *S*. *duffyi*, a eusocial species that have larger colony sizes and is geographically widespread (Cuba, Florida, Jamaica, and Panama).

In this study, we describe the complete mitochondrial genome of the eusocial sponge-dwelling snapping shrimp *S*. *microneptunus*. Specifically, we analyze the nucleotide composition and codon usage profiles of protein coding genes (PCGs), and examine selective constraints in PCGs. We also describe the secondary structure of each identified tRNA gene, and examine the putative D-loop/control region (CR). In addition, we examine the phylogenetic position of *S*. *microneptunus* among other caridean shrimps based on mitochondrial PCGs.

## Methods

### Field collection and sequencing

A single specimen collected from Barbados in 2008^13^ was used for DNA extraction and low-coverage whole genome sequencing (LC-WGS). A detailed field collection protocol has been reported previously^15^. We extracted genomic DNA using several walking legs from this alcohol-preserved specimen using the Qiagen DNeasy Tissue Kit (Qiagen). Extracted DNA was quantified using a Qubit 3.0 Fluorometer with the dsDNA HS assay (ThermoFisher Scientific) and visualized on 2% agarose gels. For LC-WGS, we provided 1,500 ng of genomic DNA to Novogene (Chula Vista, CA) for TruSeq PCR-free library preparation (Illumina) and 150 bp pair-end sequencing on an Illumina NovaSeq to obtain at least 1X coverage according to published genome size^16^. LC-WGS reads from whole-cell extraction contain a high copy number of extranuclear sequences, and it has been shown to be an efficient and economical approach to assemble complete mitochondrial genomes^17^.

### Mitochondrial genome assembly of *Synalpheus microneptunus*

The mitochondrial genome of *S*. *microneptunus* was *de novo*-assembled using the NOVOPlasty pipeline v. 1.2.3^17^. NOVOPlasty uses a seed-and-extend algorithm that assembles organelle genomes from WGS data, starting from a related or distant single ‘seed’ sequence and an optional ‘bait’ reference mitochondrial genome^17^. For assembly, we used a previously published fragment of the COI gene from *S*. *microneptunus* (GenBank accession number KJ595111) as a seed and a kmer size of 39. We did not use a bait reference mitochondrial genome considering that there are no mitochondrial genomes from closely related (congeneric) species published and available in GenBank. Nuclear mitochondrial pseudogenes are abundant in the closely related genus *Alpheus*^18^ and may affect the assembly quality, resulting in many contigs. However, the adverse effect of mitochondrial pseudogenes is likely minimal when the contigs are being circularized.

### Mitochondrial genome annotation and analysis

The newly assembled mitochondrial genome was first annotated in the MITOS web server (http://mitos.bioinf.uni-leipzig.de)^19^ using the invertebrate genetic code. Annotation curation, including start and stop codons corrections, were conducted using Expasy (https://web.expasy.org/)^20^ and MEGA X^21^. Genome visualization was conducted with OrganellarGenomeDRAW (https://chlorobox.mpimp-golm.mpg.de/OGDraw.html)^22^. Nucleotide composition and codon usage profiles of PCGs were analyzed. Nucleotide composition was estimated in MEGA X. Codon usage for each PCG was predicted using the invertebrate mitochondrial code in the Codon Usage web server (http://www.bioinformatics.org/sms2/codon_usage.html)^23^. tRNA genes were identified and their secondary structures were predicted in the software MITFI^24^ as implemented in the MITOS web server. tRNA secondary structure was visualized in the Forna web server (http://rna.tbi.univie.ac.at/forna)^25^.

We explored the selective constraints in all mitochondrial PCGs of *S*. *microneptunus*. Overall values of K_A_ (the number of nonsynonymous substitutions per nonsynonymous site: K_A_ = d_N_ = S_A_/L_A_), K_S_ (the number of synonymous substitutions per synonymous site: K_S_ = d_S_ = S_S_/L_S_), and the K_A_/K_S_ ratio (or ω or d_N_/d_S_) were estimated for each PCG in the software KaKs_calculator 2.0^26^. K_A_ and K_S_ values were based on a pairwise comparison between *S*. *microneptunus* and *Alpheus lobidens* (GenBank accession number KP276147), a species belonging to a genus sister to *Synalpheus*^27^. We chose to use *A. lobidens* for the comparison because the mitochondrial genome of this species^28^ is best described among other *Alpheus*. Next, to identify positively selected sites along the length of each examined sequence, we also calculated the values of K_A_, K_s_, and K_A_/K_S_ along sliding windows of 57 bp that ‘slipped’ every 6 bp along each PCG. The γ-MYN model^29^ was used during calculations to account for variable mutation rates across sequence sites^26^. If PCGs are under no selection, positive selective constraint (purifying selection), or diversifying selection, the K_A_/K_S_ ratio is expected to be equal to 1, >1, or <1, respectively^26^. To confirm that the observed ratios of K_A_/K_S_ were not affected by the choice of the outgroup *Alpheus* species, we repeated the above analyses with all other species of *Alpheus* for which mitochondrial genomes were available in GenBank: *A*. *bellulus*, *A*. *distinguendus*, *A*. *inopinatus*, and *A*. *randalli* (GenBank accession numbers: MH796167, NC_014883, MG551491, and MH796168, respectively).

The presence of inverted repeats in the putative D-loop/CR of *S*. *microneptunus* was explored with the ‘EMBOSS:einverted’ web server (http://www.bioinformatics.nl/cgi-bin/emboss/einverted) using the default options^30^. The presence and number of microsatellites (Simple Sequence Repeats, SSRs) were investigated with the 'Microsatellite repeats finder’ web server using the default options (http://insilico.ehu.es/mini_tools/microsatellites)^31^. The RNAstructure web server (http://rna.urmc.rochester.edu/RNAstructureWeb/Servers/Predict1/Predict1.html)^32^ was used to predict the lowest free energy secondary structure of the putative control region with particular attention to the presence of stem-loops.

Lastly, we examined the phylogenetic position of *S*. *microneptunus* among other species of caridean shrimps (Decapoda: Caridea). The newly assembled and annotated mitochondrial genome of *S*. *microneptunus* and those of a total of 63 other species of carideans available in the GenBank database were used for phylogenetic analyses conducted using the MitoPhAST pipeline v2.0^33^. Phylogenetic analyses included a total of 27 different genera in the infraorder Caridea. Outgroups included one species of lobster (*Stereomastis sculpta* [Polychelida]), two species of stenopodid shrimps (*Stenopus hispidus* and *Spongicola levigatus* [Stenopodidea]) and two species of prawns (*Penaeus vannamei* and *P*. *monodon* [Penaeoidea]). MitoPhAST extracts all 13 PCG nucleotide sequences from species available in GenBank and others provided by the user (i.e., *S*. *microneptunus*), translates each PCG nucleotide sequence to amino acids, conducts alignments for each PCG amino acid sequence using Clustal Omega^34^, removes poorly aligned regions with trimAl^35^, partitions the dataset and select best fitting models of sequence evolution for each PCG with ProtTest^36^ and uses the concatenated and partitioned PCG amino acid alignments to perform a maximum likelihood phylogenetic analysis in the software IQ-TREE^37^. The robustness of the ML tree topology was assessed by 1,000 bootstrap reiterations of the observed data. We also optimized the resulting amino acid sequence matrix under Bayesian Inference in MrBayes v3.2.7a^38^ with the same partitioning and model scheme as the ML search in IQ-TREE. Because MrBayes does not natively support one of the best fit models of molecular evolution identified with ProtTest (MtZoa), we manually implemented the substitution rate and state frequency priors from Rota-Stabelli *et al.*^39^. Our search spanned 5 million generations with four chains set to default temperature. We disregarded 25% of sampled trees as burn-in and sampled every 500 cycles. We assessed effective sample size in Tracer v 1.7^40^ and convergence of runs through the average standard deviation of split frequencies.

## Results and Discussion

**Figure 1:**
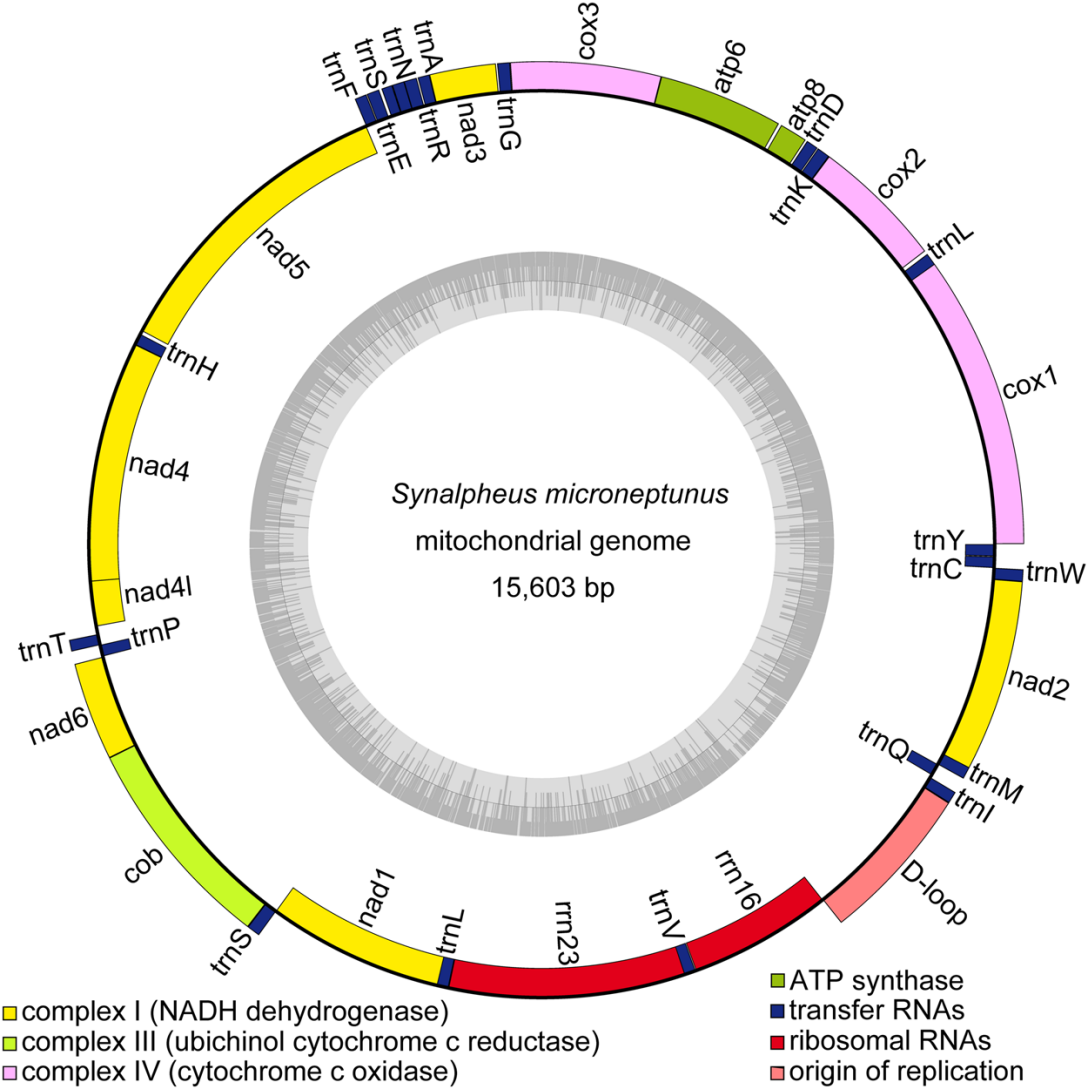
Circular genome map of *Synalpheus microneptunus* mitochondrial DNA. The annotated map depicts 13 protein-coding genes (PCGs), two ribosomal RNA genes (rrnS: 12 S ribosomal RNA and rrnL: 16 S ribosomal RNA), 22 transfer RNA (tRNA) genes, and the putative control region. The inner circle depicts GC content along the genome. The putative D-Loop/control region is not annotated. Genome assembly and initial annotation were done with NOVOPlasty^17^ and MITOS^19^, and visualized with OrganellarGenomeDRAW^22^.

Using 51,305,421 paired-end sequences (SRA: SRX6711388), we completely assembled and circularized the mitochondrial genome of *S*. *microneptunus* with a coverage of 22×(GenBank accession number MN750781). The complete mitochondrial genome of *S*. *microneptunus* was 15,603 bp in length and comprised 13 protein-coding genes (PCGs), two ribosomal RNA genes (*rrnS* [12 S ribosomal RNA] and *rrnL* [16 S ribosomal RNA]), and 22 transfer RNA (tRNA) genes. The mitochondrial genome of *S*. *microneptunus* was compact with only a few intergenic spaces and overlaps among gene junctions (Fig. 1, Table 1). Most of the PCGs and tRNA genes were encoded on the heavy strand, while only four PCGs (in order from 5′ to 3′: *nad5*, *nad4*, *nad4l*, and *nad1*), two ribosomal RNA genes and 8 tRNA genes (*trnF*, *trnH*, *trnP*, *trnL1*, *trnV*, *trnQ*, *trnC*, and *trnY*) were encoded in the light strand (Fig. 1, Table 1). A single, long intergenic space of 834 bp was assumed to be the D-loop/control region (Fig. 1, Table 1). The gene order observed in *S*. *microneptunus* is identical to that reported for most caridean shrimps^33^ and corresponds to the presumed Pancrustacean (Hexapoda + Crustacea) ground pattern^28^. Interestingly, the gene order observed in *S*. *microneptunus* is different from that reported in the closely related genus *Alpheus*^28,41–45^. However, whether or not mitochondrial gene synteny is useful to reveal genealogical relationships within the Caridea and other decapod crustaceans remains to be addressed.

**Table 1:** Arrangement and annotation of the mitochondrial genome of *Synalpheus microneptunus*.

Name	Type	Start	Stop	Strand	Length (bp)	Start	Stop	Anticodon	Inter-Genic space	Overlap
Cox1	Coding	1	1539	+	1539	ACG	TAA			5
trnL2(tta)	tRNA	1535	1601	+	67			TAA	18	
cox2	Coding	1620	2316	+	697	ATA	T		0	
trnK(aaa)	tRNA	2317	2386	+	70			TTT	3	
trnD(gac)	tRNA	2390	2454	+	65			GTC	9	
atp8	Coding	2464	2610	+	147	ATA	TAG		14	
atp6	Coding	2625	3278	+	656	ATA	TAA			1
cox3	Coding	3278	4064	+	787	ATG	T		0	
trnG(gga)	tRNA	4065	4131	+	67			TCC	6	
nad3	Coding	4138	4485	+	348	ATA	TAA			1
trnA(gca)	tRNA	4485	4547	+	63			TGC	5	
trnR(cga)	tRNA	4553	4621	+	69			TCG		2
trnN(aac)	tRNA	4620	4684	+	65			GTT	0	
trnS1(aga)	tRNA	4685	4742	+	58			TCT	13	
trnE(gaa)	tRNA	4756	4823	+	68			TTC	2	
trnF(ttc)	tRNA	4826	4889	−	64			GAA		1
nad5	Coding	4889	6586	−	1702	ATA	TAG		18	
trnH(cac)	tRNA	6605	6669	−	65			GTG		1
nad4	Coding	6669	8006	−	1337	ATA	TAG			4
nad4l	Coding	8003	8254	−	252	ATG	TAA		43	
trnT(aca)	tRNA	8298	8358	+	61			TGT		1
trnP(cca)	tRNA	8358	8422	−	65			TGG	2	
nad6	Coding	8425	8940	+	516	ATT	TAA			1
cob	Coding	8940	10074	+	1135	ATG	T		0	
trnS2(tca)	tRNA	10075	10145	+	71			TGA		2
nad1	Coding	10144	11130	−	987	ATA	TAG			10
trnL1(cta)	tRNA	11121	11186	−	66			TAG		0
rrnL	rRNA	11187	12503	−	1317	−	−			6
trnV(gta)	tRNA	12498	12562	−	65			TAC	2	
rrnS	rRNA	12565	13364	−	800	−	−		0	
CR ^Putative^		13365	14198	+	834				0	
trnI(atc)	tRNA	14199	14262	+	64			GAT	14	
trnQ(caa)	tRNA	14277	14344	−	68			TTG		1
trnM(atg)	tRNA	14344	14409	+	66			CAT		15
nad2	Coding	14395	15411	+	1017	ATA	TAA			2
trnW(tga)	tRNA	15410	15473	+	64			TCA		1
trnC(tgc)	tRNA	15473	15535	−	63			GCA	1	
trnY(tac)	tRNA	15537	15603	−	67			GTA		

In the mitochondrial genome of *S*. *microneptunus*, 12 out of the 13 PCGs exhibited conventional invertebrate and Pancrustacean mitochondrial start codons (ATA, ATG, and ATC) (Table 1). *Cox1* featured an alternative putative start codon (ACG) that was previously reported for other decapod crustaceans and references therein^46^, but was found only in a few caridean shrimps (i.e., *Nautilocaris saintlaurentae*^47^; *Macrobrachium rosenbergii*^48^). Eleven PCGs ended with a complete and conventional stop codon (TAA or TAG) (Table 1). The genes *cox2*, *cox3*, and c*ob* each terminated with an incomplete stop codon T. Truncated stop codons are often observed in crustacean mitochondrial genomes^43,49^ and are hypothesized to be completed via post-transcriptional poly-adenylation^50^.

The mitochondrial genome of *S*. *microneptunus* contained an A  +  T bias with an overall base composition of A = 36.6%, T = 28.0%, C = 23.9%, and G = 11.4% at the heavy strand. This A  +  T bias is within the known range reported for mitochondrial genomes in caridean shrimps^43^. The most frequently used codons found in the PCGs of *S*. *microneptunus* were UUA (Leu, N = 223 times used), UUU (Phe, N = 186), and AUU (Ile, N = 182). Least frequently used codons (excluding termination codons) included CCG (Pro, N = 9), CGG (Arg, N = 8), and AGC (Ser, N = 8) (Suppl. Mat. Table S1).

**Figure 2:**
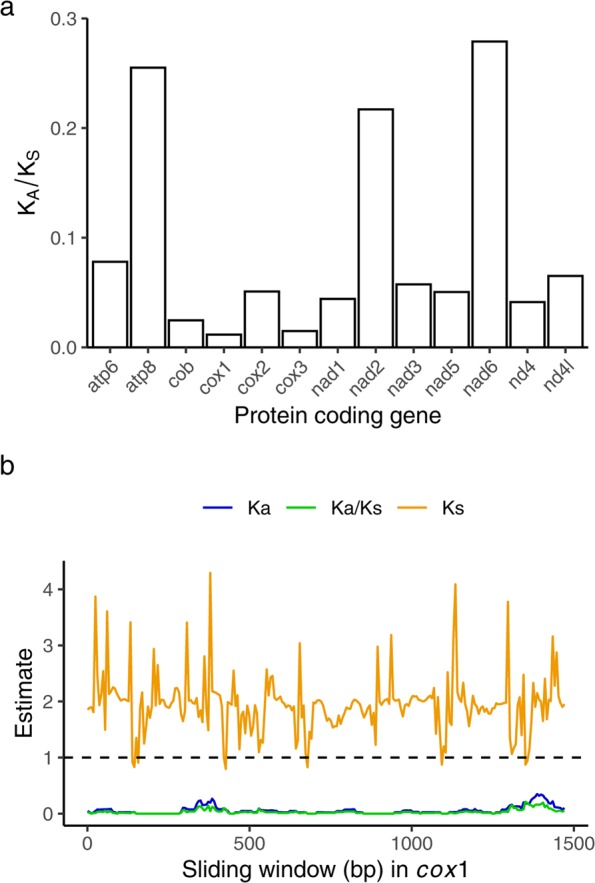
Selective pressure analysis in the protein coding genes (PCGs) of *Synalpheus microneptunus*. (**a**) Shows the K_A_/K_S_ ratios calculated using the γ-MYN model for each of the 13 PCGs. (**b**) Shows the estimate of K_A_, K_S_ and K_A_/K_S_ using a sliding window of length 57 bp and a step length of 6 bp for *cox1*. See methods and results for further details. Results of sliding-window analyses of all PCGs are shown in Suppl. Mat. Fig. S1.

All PCGs in the mitochondrial genome of *S*. *microneptunus* exhibited overall K_A_/K_S_ ratios <<1 (Fig. 2). This indicates that these PCGs are generally evolving under purifying selection. Examination of K_A_/K_S_ ratios in sliding windows across the length of each PCG further indicated that purifying selection is acting along most of the length of each PCG (Suppl. Mat. Fig. S1). The results were very similar when the above analyses were performed using other species of *Alpheus* as outgroups (Suppl. Mat. Figs. S2–S6), confirming the general pattern of purifying selection in PCGs. Selective pressure in mitochondrial PCG has been poorly studied in crustaceans but a similar pattern of widespread purifying selection in mitochondrial PCGs has been observed in other arthropods, including decapod crustaceans, and references therein^46,51^. Interestingly, regardless of *Alpheus* outgroup, the genes *atp8* and *nad6* exhibited higher K_A_/K_S_ ratios than other genes, but with values lower than 1. These two genes were also found to have higher K_A_/K_S_ ratios than other mitochondrial genes between two *Alpheus* species^42^. This suggests that selective pressures may tend to be relaxed in these genes across Alpheidae. However, broad investigations of the selective pressures in mitochondrial genes across Caridean species, or crustaceans in general, are sparse^42,47^. It is possible that eusociality may drive changes in the rate of evolution in mitochondrial genes due to prolonged longevity in the queens^52,53^, longer generation time^54,55^, and reduced effective population size^56,57^. Whether the higher K_A_/K_S_ ratios observed in a few PCGs in *S*. *microneptunus* are driven by eusociality or result from other unknown (e.g., ecological) differences between *Synalpheus* and *Alpheus* remains to be investigated in further comparative analyses.

**Figure 3:**
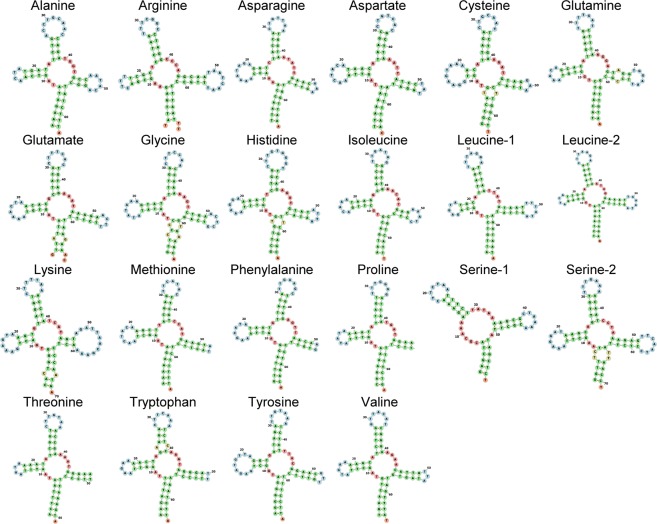
Secondary structure of tRNAs in the mitochondrial genome of *Synalpheus microneptunus* visualized in the Forna web server^25^.

The mitochondrial genome of *S*. *microneptunus* encoded tRNA genes that ranged in length from 58 (tRNA-Ser1) to 71 (tRNA-Ser2) bp. All tRNA genes, except tRNA-Ser, exhibited a standard ‘cloverleaf’ secondary structure as predicted by MIFTI (Fig. 3). In the tRNA-Ser1 gene, the stem and loop of the pseudouridine arm (T-arm) was missing. Complete (stem and loop) or partial (loop only) tRNA arm deletions are known to occur in other decapod crustaceans^46,51^, and references therein and the function of these tRNA may be complemented by elongation factors^58^.

The *rrnS* and *rrnL* genes identified in the mitochondrial genome of *S*. *microneptunus* were 800 and 1371 nucleotides long, respectively. These genes were located close to each other between tRNA-L1 and the putative D-loop/CR, but separated by tRNA-V (Fig. 1, Table 1). As shown to occur in other crustaceans, including caridean shrimps, the two genes were highly A + T biased. The overall base composition of the *rrnL* gene was A = 29.4%, T = 39.9%, C = 8.2%, and G = 22.5%. In turn, that of the *rrnS* gene was A = 29.2%, T = 38.8%, C = 8.8%, and G = 23.1%.

The 834 bp long intergenic region assumed to be the D-loop/CR was located between the 12 S ribosomal RNA and tRNA-I (Fig. 1) in *S*. *microneptunus*. The region was heavily A + T rich with an overall base composition: A = 42.0%, T = 37.5%, C = 15.2%, and G = 5.3%. Visual examination of this non-coding region revealed multiple mononucleotide adenine and thymine repeats along the entire stretch of this intergenic region. The region has an imperfect inverted repeat located in positions 176–221 and 227–275 (detected by EMBOSS:einverted) and multiple AT-rich dinucleotide and trinucleotide microsatellites along the entire stretch of the CR (detected by microsatellite Repeat Finder) (Suppl. Mat. Fig. S3). The secondary structure prediction analysis in RNAStructure (assuming 27 °C and other default options) resulted in a lowest free energy configuration (change in Gibbs free energy [ΔG] = − 104 kcal/mol) that featured various stem-loop structures interspersed along the length of the region (Suppl. Mat. Fig. S7). Only a few studies have characterized the putative D-Loop/CR in crustaceans^46^ and references therein. In some species this long non-coding region appears to be relatively well organized (i.e., in the non-decapod branchiopod genus *Daphnia*^59^ and in the decapod Chinese spiny lobster *Panulirus stimpsoni*^60^). While in other species (e.g., the Caribbean spiny lobster *Panulirus argus*^46^) and here in *S*. *microneptunus*, there is no obvious organization in the D-Loop/CR.

**Figure 4:**
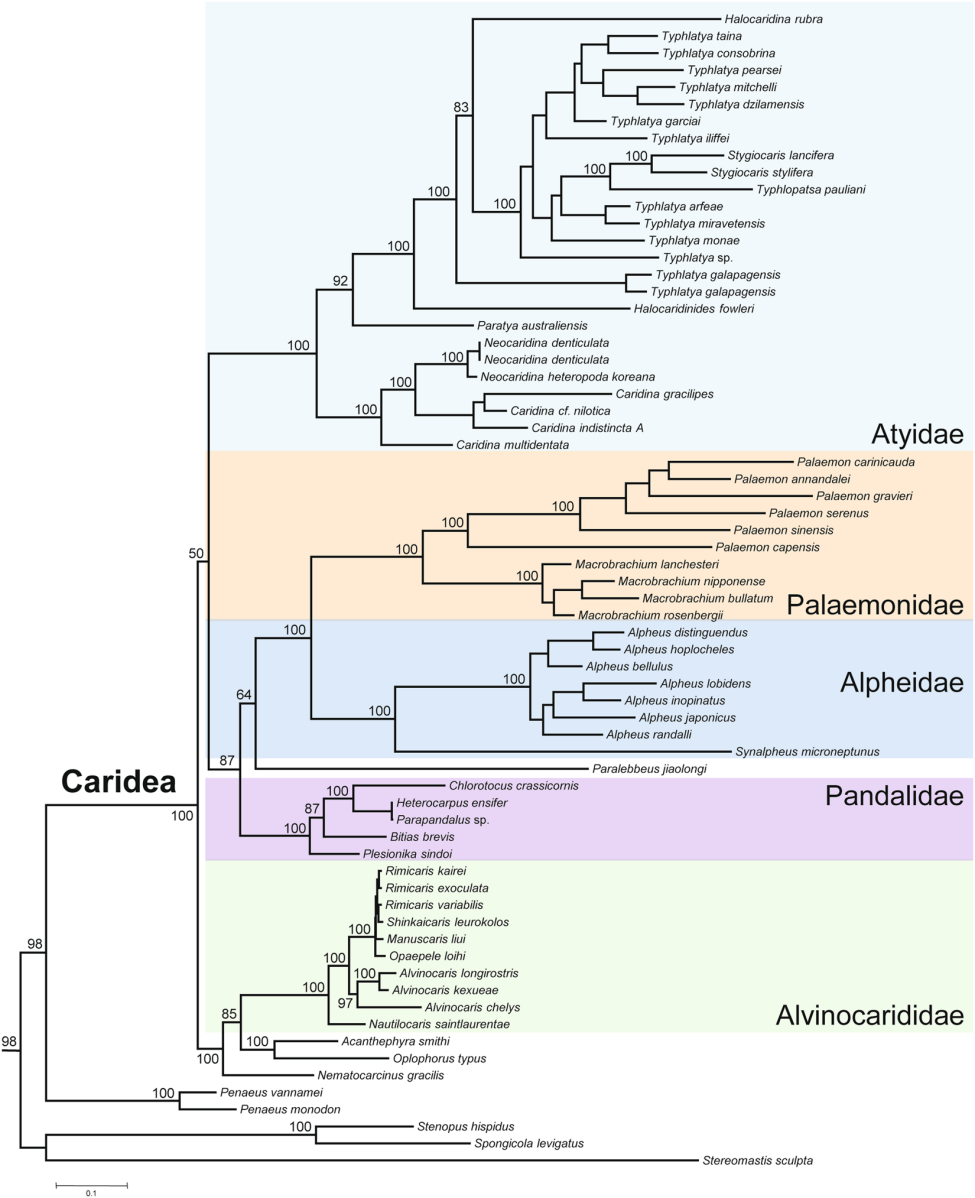
Maximum-likelihood phylogenetic tree based on the amino acid alignments of 13 protein coding genes in the mitochondrial genome of *Synalpheus microneptunus* and 43 caridean shrimps. Outgroups included one species of lobster (*Stereomastis sculpta*), two species of stenopodid shrimps (*Stenopus hispidus* and *Spongicola levigatus*) and two species of prawns (*Penaeus vannamei* and *P*. *monodon*). Number at each node represents bootstrap values. The analysis included a total of 68 terminals, 3636 amino acid characters, and 1864 parsimony informative sites. The tree was drawn using Mesquite v3.6^63^.

**Figure 5:**
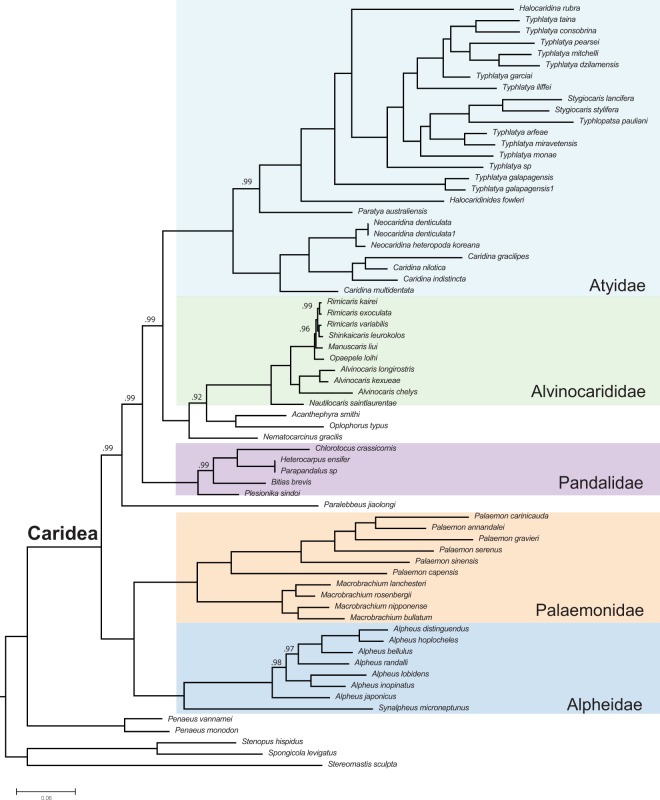
Bayesian inference phylogeny from optimization of amino acid sequences for 13 protein coding mitochondrial genes across 68 terminals. Posterior probability support indicated at nodes where values are not equal to 1.0. The tree was drawn using Mesquite v3.6^63^.

The ML and BI phylogenetic trees (68 terminals, 3636 amino acid characters, and 1864 parsimony informative sites) support the monophyly of the Caridea and placed *S*. *microneptunus* in a monophyletic clade (family Alpheidae) sister to representatives from the genus *Alpheus*. The above relationship supports the monophyly of the family Alpheidae in agreement with results from previous phylogenetic studies using a combination of partial mitochondrial and nuclear genes^61^ (Figs. 4–5) or using mitochondrial PCGs but with a more limited sample of caridean shrimps^42–44^. Additional well supported clades within the Caridea included the families Alvinocaridae, Atyidae, Palaemonidae, and Pandalidae. While the monophyly of these caridean families was supported in both ML and BI analyses, the relationships among families was found to be sensitive to optimality criteria. These differences are reflected in the low support we recovered for the node leading to Atyidae + (Palaemonidae + Alpheidae) + Pandalidae in the ML tree. Our BI analysis recovered a monophyletic (Palaemonidae + Alpheidae) clade; however all other inter-familial relationships are recovered as distinct from the ML topology, and with high support. The sister relationship herein observed between the families Palaemonidae and Alpheidae was also supported by a recent phylogenomic study^62^. The same phylogenomic analysis^62^ resolved Atyidae as the sister group to all other caridean taxa, however, none of our analyses recover this relationship. Our results suggest that mitochondrial genomes contain enough phylogenetic information to delineate monophyly of higher taxa within the Caridea (at superfamily and family levels), but the relationships among monophyletic clades may be sensitive to marker choice and reconstruction methodology.

## Conclusions

This study assembled and analyzed the first mitochondrial genome of a eusocial marine invertebrate, the caridean shrimp *S*. *microneptunus*. The complete mitochondrial genome of *S*. *microneptunus* will enhance the genomic resources in the only known group of eusocial animals in the sea and allow further investigations of the relationship between complex social behaviors (i.e., eusociality and communal living)^9^ and selective pressures and rates of molecular evolution in mitochondrial genomes.

## Supplementary information

Supplementary Information.

## Data Availability

Data is available at GenBank (MN750781 and SRX6711388).

## References

[1] Hultgren, K. M., Duffy, J. E. & Rubenstein, D. R. In Comparative Social Evolution (eds. Rubenstein, D.R. & Abbot, P.) 224–249 (Cambridge University Press, 2017).

[2] Duffy JE (1996). Eusociality in a coral-reef shrimp. Nature.

[3] Sherman PW, Lacey EA, Reeve HK, Keller L (1995). The eusociality continuum. Behav. Ecol..

[4] Chak STC, Duffy JE, Rubenstein DR (2015). Reproductive skew drives patterns of sexual dimorphism in sponge-dwelling snapping shrimps. Proc. R. Soc..

[5] Chak STC, Rubenstein DR, Duffy JE (2015). Social control of reproduction and breeding monopolization in the eusocial snapping shrimp *Synalpheus elizabethae*. The American Naturalist.

[6] Tóth E, Duffy JE (2005). Coordinated group response to nest intruders in social shrimp. Biol. Lett..

[7] Duffy JE, Morrison CL, Macdonald KS (2002). Colony defense and behavioral differentiation in the eusocial shrimp *Synalpheus regalis*. Behav. Ecol. Sociobiol..

[8] Morrison CL, Ríos R, Duffy JE (2004). Phylogenetic evidence for an ancient rapid radiation of Caribbean sponge-dwelling snapping shrimps (*Synalpheus*). Mol Phylogenet Evol.

[9] Chak, S. T. C., Duffy, J. E., Hultgren, K. M. & Rubenstein, D. R. Evolutionary transitions towards eusociality in snapping shrimps. *Nature Ecology & Evolution* **1**, 0096, 10.1038/s41559-017-0096 (2017). 10.1038/s41559-017-0096 28812668

[10] Duffy JE, Macdonald KS (2010). Kin structure, ecology and the evolution of social organization in shrimp: a comparative analysis. Proc Biol Sci.

[11] Duffy JE, Macdonald KS, Hultgren KM, Chak STC, Rubenstein DR (2013). Decline and local extinction of Caribbean eusocial shrimp. PLoS ONE.

[12] Gaynor KM (2017). Development of genome- and transcriptome-derived microsatellites in related species of snapping shrimps with highly duplicated genomes. Molecular Ecology Resources.

[13] Hultgren KM, MacDonald KS, Duffy E (2011). Sponge-dwelling snapping shrimps (Alpheidae: *Synalpheus*) of Barbados, West Indies, with a description of a new eusocial species. Zootaxa.

[14] Hultgren KM, Brandt A (2015). Taxonomy and phylogenetics of the species-complex (Decapoda: Alpheidae), with a description of two new species. J. Crust. Biol..

[15] Macdonald KS, Ríos R, Duffy JE (2006). Biodiversity, host specificity, and dominance by eusocial species among sponge-dwelling alpheid shrimp on the Belize Barrier Reef. Divers. Distrib..

[16] Jeffery NW, Hultgren K, Chak STC, Gregory TR, Rubenstein DR (2016). Patterns of genome size variation in snapping shrimp. Genome.

[17] Dierckxsens N, Mardulyn P, Smits G (2016). NOVOPlasty:de novoassembly of organelle genomes from whole genome data. Nucleic Acids Res.

[18] Williams ST, Knowlton N (2001). Mitochondrial pseudogenes are pervasive and often insidious in the snapping shrimp genus *Alpheus*. Mol. Biol. Evol.

[19] Bernt M (2013). MITOS: improved de novo metazoan mitochondrial genome annotation. Mol Phylogenet Evol.

[20] Artimo P (2012). ExPASy: SIB bioinformatics resource portal. Nucleic Acids Res.

[21] Kumar S, Stecher G, Li M, Knyaz C, Tamura K (2018). MEGA X: molecular evolutionary genetics analysis across computing platforms. Mol. Biol. Evol..

[22] Lohse M, Drechsel O, Kahlau S, Bock R (2013). OrganellarGenomeDRAW–a suite of tools for generating physical maps of plastid and mitochondrial genomes and visualizing expression data sets. Nucleic Acids Res.

[23] Stothard P (2000). The sequence manipulation suite: JavaScript programs for analyzing and formatting protein and DNA sequences. BioTechniques.

[24] Jühling F (2012). Improved systematic tRNA gene annotation allows new insights into the evolution of mitochondrial tRNA structures and into the mechanisms of mitochondrial genome rearrangements. Nucleic Acids Res.

[25] Kerpedjiev P, Hammer S, Hofacker IL (2015). Forna (force-directed. RNA): Simple and effective online RNA secondary structure diagrams. Bioinformatics.

[26] Wang D, Zhang Y, Zhang Z, Zhu J, Yu J (2010). KaKs_Calculator 2.0: A toolkit incorporating gamma-series methods and sliding window strategies. Genomics, Proteomics &. Bioinformatics.

[27] Bracken HD, De Grave S, Felder DL (2009). Phylogeny of the infraorder Caridea based on mitochondrial and nuclear genes (Crustacea: Decapoda). Decapod crustacean phylogenetics.

[28] Tan MH, Gan HM, Lee YP, Poore GCB, Austin CM (2017). Digging deeper: new gene order rearrangements and distinct patterns of codons usage in mitochondrial genomes among shrimps from the Axiidea, Gebiidea and Caridea (Crustacea: Decapoda). PeerJ.

[29] Wang, D., Wan, H.-L., Zhang, S. & Yu, J. γ-MYN: a new algorithm for estimating Ka and Ks with consideration of variable substitution rates. *Biology Direct* **4**, 20, 10.1186/1745-6150-4-20 (2009). 10.1186/1745-6150-4-20 PMC2702329 19531225

[30] Rice P, Longden I, Bleasby A (2000). EMBOSS: The European Molecular Biology Open Software Suite. Trends Genet..

[31] Bikandi J, Millan RS, Rementeria A, Garaizar J (2004). In silico analysis of complete bacterial genomes: PCR, AFLP-PCR and endonuclease restriction. Bioinformatics.

[32] Reuter JS, Mathews DH (2010). RNAstructure: software for RNA secondary structure prediction and analysis. BMC Bioinformatics.

[33] Tan MH, Gan HM, Schultz MB, Austin CM (2015). MitoPhAST, a new automated mitogenomic phylogeny tool in the post-genomic era with a case study of 89 decapod mitogenomes including eight new freshwater crayfish mitogenomes. Mol. Phylogen. Evol.

[34] Sievers F (2014). Fast, scalable generation of high-quality protein multiple sequence alignments using Clustal Omega. Mol. Syst. Biol..

[35] Capella-Gutierrez S, Silla-Martinez JM, Gabaldon T (2009). trimAl: a tool for automated alignment trimming in large-scale phylogenetic analyses. Bioinformatics.

[36] Abascal F, Zardoya R, Posada D (2005). ProtTest: selection of best-fit models of protein evolution. Bioinformatics.

[37] Nguyen L-T, Schmidt HA, Von Haeseler A, Minh BQ (2015). IQ-TREE: A fast and effective stochastic algorithm for estimating maximum-likelihood phylogenies. Mol. Biol. Evol..

[38] Ronquist F (2012). MrBayes 3.2: efficient Bayesian phylogenetic inference and model choice across a large model space. Syst. Biol..

[39] Rota-Stabelli O, Yang Z, Telford MJ (2009). MtZoa: A general mitochondrial amino acid substitutions model for animal evolutionary studies. Mol. Phylogen. Evol..

[40] Rambaut A, Drummond AJ, Xie D, Baele G, Suchard MA (2018). Posterior summarization in Bayesian phylogenetics using Tracer 1.7. Syst. Biol..

[41] Qian G (2011). Two new decapod (Crustacea, Malacostraca) complete mitochondrial genomes: bearings on the phylogenetic relationships within the Decapoda. Zool. J. Linn. Soc..

[42] Shen X, Li X, Sha Z, Yan B, Xu Q (2012). Complete mitochondrial genome of the Japanese snapping shrimp *Alpheus japonicus* (Crustacea: Decapoda: Caridea): Gene rearrangement and phylogeny within Caridea. Science China Life Sciences.

[43] Tan MH (2019). Comparative mitogenomics of the Decapoda reveals evolutionary heterogeneity in architecture and composition. Scientific Reports.

[44] Zhong S, Zhao Y, Zhang Q (2019). The complete mitochondrial genome of *Alpheus hoplocheles* (Decapoda: Alpheidae). Mitochondrial DNA Part B.

[45] Wang Q (2020). Characterization and comparison of the mitochondrial genomes from two Alpheidae species and insights into the phylogeny of Caridea. Genomics.

[46] Baeza JA (2018). The complete mitochondrial genome of the Caribbean spiny lobster *Panulirus argus*. Scientific Reports.

[47] Kim S-J, Pak SJ, Ju S-J (2015). Mitochondrial genome of the hydrothermal vent shrimp *Nautilocaris saintlaurentae* (Crustacea: Caridea: Alvinocarididae). Mitochondrial DNA.

[48] Miller AD, Murphy NP, Burridge CP, Austin CM (2005). Complete Mitochondrial DNA Sequences of the Decapod Crustaceans *Pseudocarcinus gigas* (Menippidae) and *Macrobrachium rosenbergii* (Palaemonidae). Mar. Biotechnol..

[49] Ivey JL, Santos SR (2007). The complete mitochondrial genome of the Hawaiian anchialine shrimp *Halocaridina rubra* Holthuis, 1963 (Crustacea: Decapoda: Atyidae). Gene.

[50] Beckenbach A (2009). Numts and mitochondrial pseudogenes. Myrmecological News.

[51] Li T (2016). A mitochondrial genome of Rhyparochromidae (Hemiptera: Heteroptera) and a comparative analysis of related mitochondrial genomes. Scientific Reports.

[52] Keller L, Genoud M (1997). Extraordinary lifespans in ants: a test of evolutionary theories of ageing. Nature.

[53] Schmidt CM, Jarvis JUM, Bennett NC (2013). The long-lived queen: reproduction and longevity in female eusocial Damaraland mole-rats (*Fukomys damarensis*). Afr. Zool..

[54] Thorne, B. L., Breisch, N. L. & Haverty, M. I. Longevity of kings and queens and first time of production of fertile progeny in dampwood termite (Isoptera; Termopsidae; *Zootermopsis*) colonies with different reproductive structures. *Journal of Animal Ecology* **71**, 1030–1041, 10.1046/j.1365-2656.2002.00666.x (2002).

[55] Ingram CM, Troendle NJ, Gill CA, Braude S, Honeycutt RL (2015). Challenging the inbreeding hypothesis in a eusocial mammal: population genetics of the naked mole-rat, Heterocephalus glaber. Mol. Ecol..

[56] Romiguier J (2014). Population genomics of eusocial insects: the costs of a vertebrate-like effective population size. J. Evol. Biol..

[57] Bromham L, Leys R (2005). Sociality and the rate of molecular evolution. Mol. Biol. Evol..

[58] Watanabe Y-I, Suematsu T, Ohtsuki T (2014). Losing the stem-loop structure from metazoan mitochondrial tRNAs and co-evolution of interacting factors. Frontiers in Genetics.

[59] Kuhn K, Streit B, Schwenk K (2008). Conservation of structural elements in the mitochondrial control region of *Daphnia*. Gene.

[60] Liu Y, Cui Z (2011). Complete mitochondrial genome of the Chinese spiny lobster Panulirus stimpsoni (Crustacea: Decapoda): genome characterization and phylogenetic considerations. Molecular Biology Reports.

[61] Palero F, Crandall KA, Abelló P, Macpherson E, Pascual M (2009). Phylogenetic relationships between spiny, slipper and coral lobsters (Crustacea, Decapoda, Achelata). Mol. Phylogen. Evol.

[62] Wolfe JM (2019). A phylogenomic framework, evolutionary timeline and genomic resources for comparative studies of decapod crustaceans. Proc. R. Soc..

[63] Mesquite: a modular system for evolutionary analysis v. 3.61 (2019).

