# Supplementary material for: The complete mitochondrial genome of the eusocial sponge-dwelling snapping shrimp Synalpheus microneptunus
Source: Sci Rep. 2020 May 8;10:7744. doi: 10.1038/s41598-020-64269-w (PMC7210941; doi:10.1038/s41598-020-64269-w)
Supplement: Supplementary file 1 — Supplementary Information. [file 41598_2020_64269_MOESM1_ESM.docx]

**The complete mitochondrial genome of the eusocial sponge-dwelling snapping shrimp *Synalpheus microneptunus***

Solomon T. C. Chak^1^_,_ Phillip Barden^1*^, J. Antonio Baeza^2,3,4*^

^1^Department of Biological Sciences, New Jersey Institute of Technology, Newark, NJ 07102, USA. ^2^Department of Biological Sciences, 132 Long Hall, Clemson University, Clemson, SC, 29634, USA. ^3^Smithsonian Marine Station at Fort Pierce, 701 Seaway Drive, Fort Pierce, Florida, 34949, USA. ^4^Departamento de Biología Marina, Facultad de Ciencias del Mar, Universidad Católica del Norte, Larrondo 1281, Coquimbo, Chile. Correspondence and requests for materials should be addressed to J.A.B. (email: jbaezam@clemson.edu) or S.T.C.C. (email: tchak@njit.edu).

* equal co-authorship

**Supplementary Materials**

**Table S1**. Codon usage analysis of PCGs in the mitochondrial genome of *Synalpheus microneptunus*.

**Figure S1.** Selection analyses using sliding windows along each of the 13 protein coding genes.

**Figure S2.** Selection analyses the protein coding genes (PCGs) of *Synalpheus microneptunus* against four alternative *Alpheus* outgroup species.

**Figure S3**. Selection analysis along sliding windows of each PCGs of *Synalpheus microneptunus* against *Alpheus bellulus.*

**Figure S4**. Selection analysis along sliding windows of each PCGs of *Synalpheus microneptunus* against *Alpheus distinguendus.*

**Figure S5**. Selection analysis along sliding windows of each PCGs of *Synalpheus microneptunus* against *Alpheus inopinatus.*

**Figure S6**. Selection analysis along sliding windows of each PCGs of *Synalpheus microneptunus* against *Alpheus randalli.*

**Figure S7.** Predicted secondary structure of the putative control region in *Synalpheus microneptunus*.

**Supplementary Table S1**. Codon usage analysis of PCGs in the mitochondrial genome of *Synalpheus microneptunus*.

| AA | Codon | N | /1000 | Freq | AA | Codon | N | /1000 | Freq |
| --- | --- | --- | --- | --- | --- | --- | --- | --- | --- |
| Ala | GCG | 12 | 3.24 | 0.05 | Pro | CCG | 9 | 2.43 | 0.06 |
|  | GCA | 48 | 12.98 | 0.21 |  | CCA | 59 | 15.95 | 0.4 |
|  | GCT | 97 | 26.23 | 0.43 |  | CCT | 43 | 11.63 | 0.29 |
|  | GCC | 69 | 18.66 | 0.31 |  | CCC | 37 | 10.01 | 0.25 |
| Cys | TGT | 31 | 8.38 | 0.74 | Gln | CAG | 21 | 5.68 | 0.28 |
|  | TGC | 11 | 2.97 | 0.26 |  | CAA | 55 | 14.87 | 0.72 |
| Asp | GAT | 46 | 12.44 | 0.6 | Arg | CGG | 8 | 2.16 | 0.13 |
|  | GAC | 31 | 8.38 | 0.4 |  | CGA | 30 | 8.11 | 0.5 |
| Glu | GAG | 27 | 7.3 | 0.36 |  | CGT | 12 | 3.24 | 0.2 |
|  | GAA | 47 | 12.71 | 0.64 |  | CGC | 10 | 2.7 | 0.17 |
| Phe | TTT | 186 | 50.3 | 0.68 | Ser | AGG | 30 | 8.11 | 0.08 |
|  | TTC | 87 | 23.53 | 0.32 |  | AGA | 64 | 17.31 | 0.18 |
| Gly | GGG | 68 | 18.39 | 0.27 |  | AGT | 22 | 5.95 | 0.06 |
|  | GGA | 98 | 26.5 | 0.39 |  | AGC | 8 | 2.16 | 0.02 |
|  | GGT | 47 | 12.71 | 0.19 |  | TCG | 15 | 4.06 | 0.04 |
|  | GGC | 38 | 10.28 | 0.15 |  | TCA | 85 | 22.99 | 0.23 |
| His | CAT | 30 | 8.11 | 0.35 |  | TCT | 101 | 27.31 | 0.28 |
|  | CAC | 56 | 15.14 | 0.65 |  | TCC | 40 | 10.82 | 0.11 |
| Ile | ATT | 182 | 49.22 | 0.64 | Thr | ACG | 16 | 4.33 | 0.07 |
|  | ATC | 101 | 27.31 | 0.36 |  | ACA | 63 | 17.04 | 0.28 |
| Lys | AAG | 20 | 5.41 | 0.24 |  | ACT | 80 | 21.63 | 0.36 |
|  | AAA | 63 | 17.04 | 0.76 |  | ACC | 65 | 17.58 | 0.29 |
| Leu | TTG | 101 | 27.31 | 0.17 | Val | GTG | 43 | 11.63 | 0.17 |
|  | TTA | 223 | 60.3 | 0.38 |  | GTA | 85 | 22.99 | 0.33 |
|  | CTG | 13 | 3.52 | 0.02 |  | GTT | 94 | 25.42 | 0.37 |
|  | CTA | 146 | 39.48 | 0.25 |  | GTC | 32 | 8.65 | 0.13 |
|  | CTT | 73 | 19.74 | 0.12 | Trp | TGG | 21 | 5.68 | 0.21 |
|  | CTC | 35 | 9.46 | 0.06 |  | TGA | 80 | 21.63 | 0.79 |
| Met | ATG | 57 | 15.41 | 0.28 | Tyr | TAT | 96 | 25.96 | 0.69 |
|  | ATA | 149 | 40.29 | 0.72 |  | TAC | 44 | 11.9 | 0.31 |
| Asn | AAT | 61 | 16.5 | 0.48 | End | TAG | 4 | 1.08 | 0.4 |
|  | AAC | 67 | 18.12 | 0.52 |  | TAA | 6 | 1.62 | 0.6 |

**Figure S1.** Selection analyses using sliding windows along each of the 13 protein coding genes.

**Figure S2.** Selection analyses the protein coding genes (PCGs) of *Synalpheus microneptunus* against four alternative *Alpheus* outgroup species. K_A_/K_S_ ratios were calculated using the γ-MYN model for each PCGs.

**Figure S3**. Selection analysis along sliding windows of each PCGs of *Synalpheus microneptunus* against *Alpheus bellulus.* K_A_/K_S_ were estimated for each sliding window of 57 bp that were 6 bp apart.

**Figure S4**. Selection analysis along sliding windows of each PCGs of *Synalpheus microneptunus* against *Alpheus distinguendus.* K_A_/K_S_ were estimated for each sliding window of 57 bp that were 6 bp apart.

**Figure S5**. Selection analysis along sliding windows of each PCGs of *Synalpheus microneptunus* against *Alpheus inopinatus.* K_A_/K_S_ were estimated for each sliding window of 57 bp that were 6 bp apart.

**Figure S6**. Selection analysis along sliding windows of each PCGs of *Synalpheus microneptunus* against *Alpheus randalli.* K_A_/K_S_ were estimated for each sliding window of 57 bp that were 6 bp apart.

**Figure S7.** Predicted secondary structure of the putative control region in *Synalpheus microneptunus* using the RNAstructure web server ^1^.

**References**

1 Reuter, J. S. & Mathews, D. H. RNAstructure: software for RNA secondary structure prediction and analysis. *BMC Bioinformatics* **11**, 129, doi:10.1186/1471-2105-11-129 (2010).
